# Insights into disability and psycho-social care of patients with inflammatory bowel disease

**DOI:** 10.3389/fmed.2024.1416054

**Published:** 2024-05-28

**Authors:** Olga Maria Nardone, Giulio Calabrese, Alessia La Mantia, Rossella Caso, Anna Testa, Fabiana Castiglione

**Affiliations:** ^1^Department of Public Health, University of Naples Federico II, Naples, Italy; ^2^Department of Clinical Medicine and Surgery, University of Naples Federico II, Naples, Italy

**Keywords:** disability, holistic care, bowel urgency, fatigue, sexual dysfunction, fertility

## Abstract

In recent years, the concept of disability has increasingly garnered attention as a crucial long-term target of inflammatory bowel disease (IBD) management. The treatment paradigm has changed dramatically from full control of the disease (clinical and endoscopic remission) toward physical and emotional well-being with the goal of preventing disability and normalizing quality of life. However, in certain cases, despite achieving good disease control, patients may still experience symptoms associated with disability, and reduced emotional wellness. These symptoms can significantly impact various biopsychosocial factors, including interpersonal relationships, educational or work-related activities, body image, and sexual functioning. Nevertheless, they often remain overlooked in the context of IBD care. In this narrative review, we aim to shed light on the burden of certain disability-related symptoms such as bowel urgency, sexual dysfunction, impaired fertility and fatigue, emphasizing the importance of acknowledging and validating them in a clinical setting. There is a demanding need for comprehensive care for IBD patients, with IBD clinicians being mindful of the psychosocial challenges faced by their patients. Providing timely and appropriate management of these challenges alongside IBD treatment is key to achieving holistic remission and enhancing the overall quality of life while reducing disability.

## 1 Introduction

Crohn’s disease (CD) and ulcerative colitis (UC), belonging to the group of inflammatory bowel diseases (IBD), are chronic relapsing inflammatory conditions affecting the gastrointestinal tract (1) with incidence rising among pediatric populations and in developing nations (2, 3). The lifelong duration of IBD can lead to progressive bowel damage and disability, significantly impacting patients’ social, physical, and psychological aspects and directly influencing their overall well-being (4, 5).

In recent years, the concept of disability has increasingly garnered attention as a crucial aspect of disease management (6, 7). In 2021, the Selecting Therapeutic Targets in Inflammatory Bowel Disease (STRIDE) Initiative, led by the International Organization for the Study of Inflammatory Bowel Disease (IOIBD), identified the absence of disability as a key long-term goal for IBD patients, alongside endoscopic healing and normalization of quality of life (8).

More recently, the concept of treating to target has undergone further evolution. The target now encompasses not only the control of inflammation but also the promotion of emotional wellness (9). Consequently, therapeutic decisions may also be driven by individuals’ changing needs and based on what impacts their quality of life (HRQOL) and mitigates disability (10). Remarkably, disability differs from HRQOL, because it describes the impact of an illness on a person’s participation in life activities, reflecting an interaction between features of a person’s body and features of the society in which they live, while QOL refers to how one feels about these limitations and restrictions (11). The gap between physical and emotional wellness remains evident, with unmet needs and unaddressed aspects of psychosocial IBD care still prevalent (12, 13). Patients often express frustration with ongoing challenging symptoms, including fatigue, depression, anxiety, sexual dysfunction and emotional wellness; these can be discordant with inflammation and not optimally addressed by clinicians due to a lack of time, resources and awareness. Assessing psycho-social symptoms can be quite challenging due to their subjective nature. Accordingly, in an attempt to quantify disability, the ‘IBD Disability Index’ (IBD-DI) was developed in collaboration with the WHO (14). This is a physician-assisted questionnaire evaluating different domains of disability of IBD patients, such as overall health, sleep, energy, interpersonal relationships and disease-related features (e.g., bowel urgency, bleeding and arthralgia). Based on this, subsequently, Ghosh et al. (15) have developed a shortened and patient-friendly version of IBD-DI named IBD-disk for clinical use. Noteworthy, these tools can help promptly recognize symptoms, psychological comorbidities and emotional wellness by proactive assessment, with the overall goal of symptom control and emotional wellness.

Besides, patients who are objectively more disabled may have more difficulties accepting their disease. In this context, IBD-disk might be useful for identifying areas requiring intervention to target disease acceptance. In a recent cross-sectional study by Teugels, disease acceptance was more strongly associated with disability, especially for the domains of energy and negative emotions in patients whose disease was in clinical remission compared with those with active disease (16). Noticeably, patients who exhibit higher levels of disease acceptance may demonstrate greater openness to re-conceptualizing or re-prioritizing what holds value in their lives. This adaptive approach can help mitigate the perception of the disease as a constant threat to their core values or goals (6, 8).

**Figure 1:**
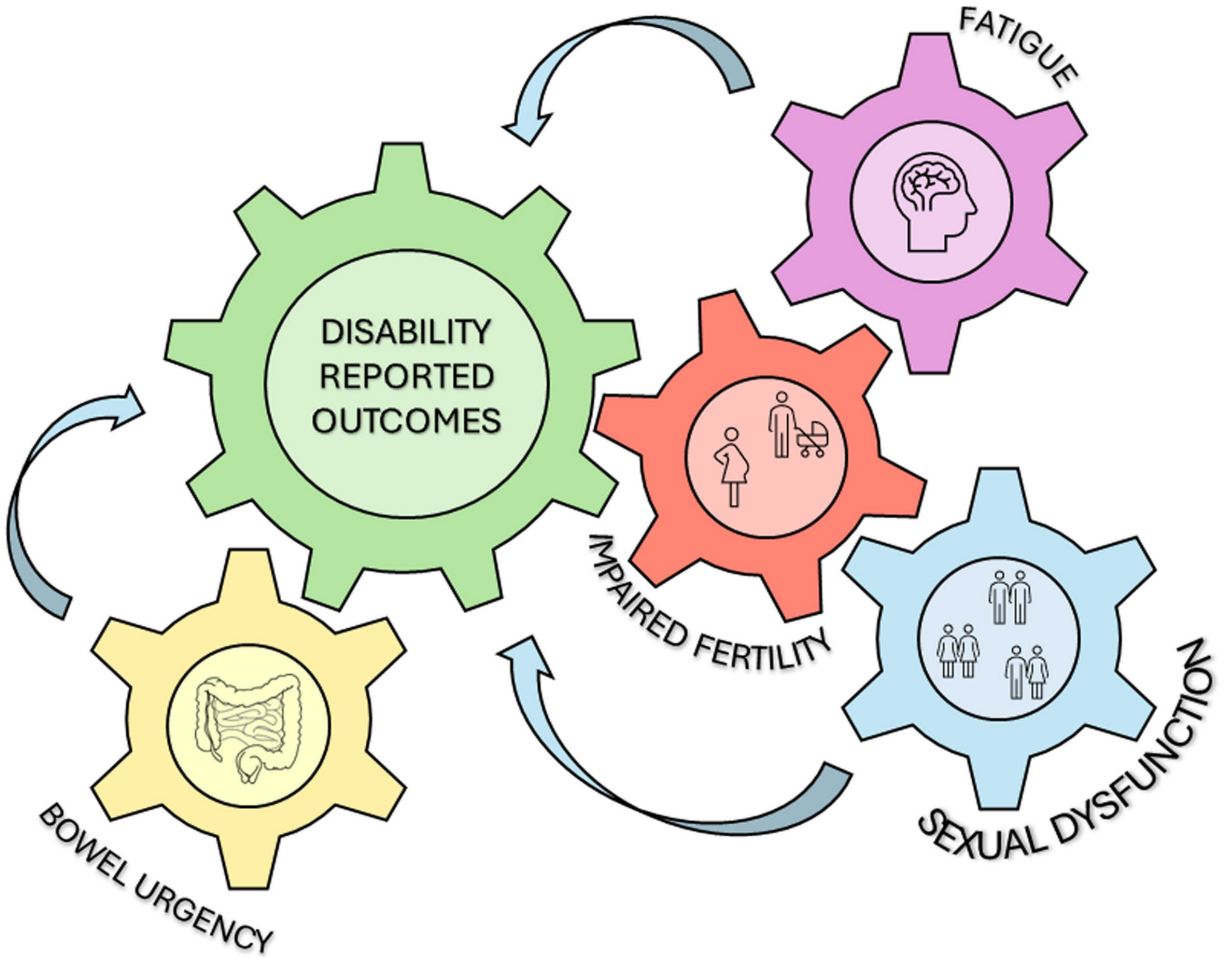
Overview of main disability associated symptoms assessed in the review.

However, there is a demanding need for comprehensive care for IBD patients, with IBD clinicians being mindful of the psychosocial challenges faced by their patients. A fully integrated approach, alongside disease control, to address mental, physical, and emotional well-being for all patients should be incorporated to achieve the target of healing from both medical and patient perspectives, ensuring that patients are treated as whole individuals. Consistently, we conducted a narrative review to evaluate the prevalent disability-related symptoms such as bowel urgency, sexual dysfunction, impaired fertility and fatigue aiming to underscore the importance of routinely recognizing and integrating the assessment of these symptoms into daily practice (Figure 1). This emphasizes the need for holistic patient care strategies.

## 2 Methods

A systematic literature search has been performed from inception to February 2024 using PubMed and Embase. We combined keywords and Medical Subject Headings (MeSH) as reported below: “Inflammatory Bowel Disease,” “IBD,” Crohn’s Disease,” “CD,” “ulcerative colitis,” “UC,” “disability,” “Quality of life,” “IBD Disk,” “IBD Disability Index,” “IBD DI,” “Sexual dysfunction,” “fatigue,” “bowel urgency,” “rectal urgency,” “psychosocial care,” “holistic care,” “PRO,” “patient’s reported outcomes.” Two authors (GC and ALM) screened papers first by title and then by abstract. Disagreement was resolved by consensus. Finally, we included 73 sources.

### 2.1 Bowel urgency

Bowel urgency (BU) contributes significantly to disease-related disability and severely impacts patients’ well-being, which, in turn, can exacerbate IBD clinical course (17). This symptom, characterized by the sudden and immediate need to have a bowel movement, is a bothersome and impactful aspect of UC and CD, distinct from the symptom of increased stool frequency and incontinence. In cross-sectional and observational studies, more than 80% of patients with UC and 60 to 74% of patients with CD experience BU, with 25–50% of patients experiencing BU at least once daily (18–24). However, BU is often under-recognized and not discussed by patients. Only 30% of patients from the United States and 43% from Europe in the CONFIDE study felt comfortable discussing BU with their healthcare providers (HCP), commonly due to embarrassment (25). Additionally, BU may not be adequately investigated by HCPs due to a lack of awareness of validated tools and/or knowledge of the importance of assessing BU. In a recent international patient and physician survey, patients with IBD reported rectal urgency with the need to use the toilet as a top symptom impacting QOL, which was rated as lower-impact by physicians. The results highlight the need to implement the knowledge and assessment of BU (23).

Remarkably, BU contributes to considerable psychological stress due to various situations involving symptoms of fecal incontinence, which means involuntary loss of liquid or solid stool, fear of using public toilets, and adaptive behaviors, such as bathroom mapping (knowing the location of the nearest bathroom), wearing an adult diaper or carrying a cleanup kit. Such stress impacts on patients’ well-being, which in turn can aggravate IBD symptoms (26).

The mechanism underlying BU is intricate and multifaceted, characterized by a complex interplay of motility, structural alterations, and sensory processing (27). Initially, motility can undergo alterations, resulting in heightened transit through the sigmoid-rectum segment and intensified propulsive pressure waves, thus exacerbating the sensation of urgency. Furthermore, the modified function of the rectal wall leads to reduced distensibility, diminishing rectal capacity and causing fecal matter to accumulate in an inadequately accommodating rectum. Consequently, this generates heightened pressure from smaller volumes, intensifying the sense of BU. Additionally, the anal sphincter may exhibit “fatigability,” further contributing to the urgency sensation. Chronic inflammation can also induce structural changes, such as thickening of the muscularis mucosae and fibrosis in the submucosa, which decrease the compliance of the rectal wall, exacerbating urgency-related symptoms. Alongside these physiological alterations, increased visceral sensitivity, mediated through the brain-gut axis, amplifies the perception of urgency (27).

Evolving evidence suggests BU is associated with decreased HRQoL in patients with UC or CD and future risk of hospitalizations, use of corticosteroids and colectomy for patients with UC (28, 29). The recently updated Food and Drug Administration (FDA) guidelines for UC and CD encourage the exploration of BU by healthcare providers in routine clinical examinations and assessment of BU and bowel urgency-related accidents must be considered in clinical trials (30, 31). Consequently, several PRO tools have been developed, including the 29-item Symptoms and Impacts Questionnaire for UC, the UC Patient-Reported Outcomes Signs and Symptoms measure, the Patient-Reported Outcome-Ulcerative Colitis diary and the Urgency NRS (26). The Urgency NRS is easily understood by patients because it does not depend on specific descriptors, which may not be suitable to all patients due to the different personal experiences of BU. Indeed, patients with UC prefer a BU scale that distinguishes levels of severity instead of a binary “yes/no” response (27). The Urgency NRS is a single-item PRO measure that includes a 0–10 scale, with 0 representing no BU and 10 representing the worst possible BU. Patients report the severity of BU over the prior 24 h, with weekly average scores calculated as the mean score over 7 days. Higher scores indicate worse BU severity (32). A sub-analysis from a multicenter, randomized, placebo-controlled phase 3 trial in adults with moderately to severely active UC (NCT03518086) has shown that a ≥ 3-point improvement in the Urgency NRS score reflects a significant enhancement in bowel urgency, while an Urgency NRS score of ≤1 point represents a threshold for bowel urgency remission closely associated with clinical, endoscopic, and histologic remission (33). However, despite being easily understood, the multidimensional nature of BU implies that multiple aspects of bowel urgency should be considered in developing bowel urgency-specific PRO measures.

The inflammatory component of BU is responsive to treatment, so it can be used as a measure of disease control in active disease. Treatment with upadacitinib, a Janus kinase inhibitor, has been associated with significant improvements in BU alongside the achievement of clinical response and remission. Recently, mirikizumab, a monoclonal antibody targeting the p19 subunit of interleukin 23, has also demonstrated effectiveness in reducing BU (34). A significantly greater proportion of patients treated with mirikizumab reported no BU at week 12 versus those receiving the placebo, and this improvement was sustained through week 52 (35). Besides, the management of BU can be more complicated when independent of IBD disease activity. In that situation, supportive measures include avoiding triggering foods, biofeedback and exercises of the pelvic floor and anal sphincter can mitigate BU (27). However, in addition to assessing rectal bleeding and stool frequency, BU should be included as one of the outcome measures utilized in clinical trials, drug registration processes, and real-world studies to evaluate treatment efficacy.

### 2.2 Sexual dysfunction

The quality of sexual life represents a significant component of QoL and disability in IBD. Sexual dysfunction (SD) was defined by the 2nd International Consultation on Sexual Medicine as the “diminished or absent feelings of sexual interest or desire, absent sexual thoughts or fantasies, and a lack of responsive desire” (36). This concept manifests differently in males and females: female SD often presents as a lack of genital/subjective arousal or sexual desire, while in males it is characterized mainly by erectile dysfunction and premature/early ejaculation. Several factors, such as gender, active disease, psychosocial factors, pelvic disorders, extraintestinal manifestations and surgery, may contribute to SD development in IBD.

In the IMPACT study (37), a large survey conducted across Europe aimed at understanding the impact of IBD on patients’ lives, 40% of patients reported that disease activity negatively affected intimate relationships. In a recent meta-analysis conducted by Chen et al. (38) on 11 studies, the risk of SD in male patients with IBD was significantly higher than in individuals without IBD (OR = 1.61, 95% CI: 1.17 to 2.23, *p* = 0.004). Similarly, female patients with IBD had a significantly higher risk of SD compared to healthy controls (OR = 2.28, 95% CI: 1.54 to 3.37, *p* < 0.0001).

The prevalence of SD in female patients with IBD tends to be higher than in male patients, with rates ranging from 21.1 to 48.2% in men and from 51.0 to 73.7% in women (38). The reason behind this finding is unclear; however, women may be more concerned than men about intimacy, sexual performance and relationship issues because of their disease. Additionally, women are more likely to develop psychological illness, particularly depression, which is considered the most consistent independent risk factor for SD (39).

The strong connection between SD and mental health issues is likely bidirectional, with psychological disorders negatively impacting SD, and conversely, SD exacerbating mental health problems (40).

Of note, a previous study showed that treating anxious or depressed patients with IBD with antidepressants for 6 months resulted in improved sexual functioning, suggesting a close relationship. However, future prospective studies with a longer follow-up period are needed to confirm this data.

Pires et al. (41) recently conducted a cross-sectional survey and identified SD, body image distortion and fatigue as predictors of impaired QOL (*p* = 0.007, *p* = 0.001 and *p* = 0.004, respectively). Therefore, targeted intervention addressing SD may improve QoL and disability.

Additionally, extraintestinal manifestations, including not only joint pain but also hidradenitis suppurativa and vulvovaginal CD, can also contribute to SD development. Boyd et al. (40) had indeed evaluated the psychological impact of vaginal lesions caused by CD or hidradenitis suppurativa on IBD patients, resulting in the development of SD, primarily due to vulvodynia and impaired body image.

Data regarding the impact of surgery on SD development are controversial. Despite the potential risks related to the J-pouch, literature data show a decrease in SD rates in patients who have undergone this surgical procedure (42, 43). Conversely, ostomy creation has been found to be associated with SD, possibly due to impaired body image (44, 45). A prospective evaluation of sexual function and QoL after ileal pouch-anal anastomosis by Davies RJ (43) demonstrated that 52% of women experienced a decrease in sense of sexual attractiveness following ileostomy formation, while 60% of women felt less desirable. As a result, SD directly influences HRQoL (41, 46).

Finally, limited data exist on the impact of IBD on sexual dysfunction among sexual minorities and members of the LGBTQ (lesbian, gay, bisexual, transgender, and queer) (47). Practitioners should be aware that people exist outside of the heteronormative realm and create an environment of openness that indicates to all patients with IBD that they are well-accepted and free to talk about their sexual activity.

### 2.3 Impaired fertility

Closely related to sexual dysfunction is the fear of infertility which constitutes a significant psychological burden on women with IBD, and on couples especially, since IBDs have their peak incidence during reproductive age (48). Despite patients with inactive IBD and with no prior pelvic surgery having similar fertility rates compared to the general population (49), patients seem to have fewer children due to voluntary childlessness (50), decreased libido or dyspareunia, anxiety regarding adverse pregnancy outcomes and medication safety, concerns about disease transmission, and fears of being unable to care for a child (51).

Active disease can affect fertility in both females and males, as chronic pelvic inflammatory status, inflammation of the peritoneum and perineal disease can be responsible for abdominal adherences, tubal damage and sexual dysfunctions, thus decreasing fertility rates (52). In addition, a long-standing, active and colonic disease (53–55), may decrease the ovarian reserve in female patients.

Regarding the relation between fertility and surgery, consolidated evidence suggests that female patients with IBD may experience decreased fertility rates after undergoing ileal pouch-anal anastomosis (IPAA) via laparotomy compared to those who underwent the same surgery in the laparoscopic approach, likely due to a higher risk of pelvic adhesions (56, 57). Furthermore, IPAA could affect fertility in men too by causing retrograde ejaculation or erectile dysfunction (49).

Remarkably, different studies have explored the burden of the disease on dietary habits, which are crucial not only for IBD activity but also for fertility rates and fetal development during pregnancy. Fertility rates can be affected by micronutrient deficiencies through different mechanisms. The ovulation process, oocyte quality, implantation and embryogenesis are supported by an adequate concentration of folate, zinc, vitamin A, iron and B vitamins (58), as well as vitamin D, which is essential for the steroidogenesis of sex hormones. Moreover, malnutrition can affect fetal development, especially during placental development, since it can lead to an increased probability of inadequate Gestational Weight Gain (GWG) (59), which has been demonstrated to correlate with a higher risk of preterm birth or Small for Gestational Age (SGA) babies (60). Accurate counseling during the pre-conceptual period is the initial step in treating infertility, together with nutritional status optimization and disease control through adequate therapy. Couples experiencing infertility should be referred to a specialized center to identify causes other than the disease itself. In certain situations, Assisted Reproductive Technology (ART) may be proposed. However, research indicates that women with UC and CD have a lower likelihood of achieving a live birth per ART treatment cycle compared to women without IBD. Additionally, UC patients appear to have an elevated risk of preterm birth, while CD patients who have undergone surgery exhibit a notable reduction in live birth rates within 18 months following the commencement of ART compared to non-IBD women (61).

Finally, several studies have shown an association between voluntary childlessness and a low level of knowledge and awareness about the disease (62), indicating that education strategies and patient counseling could play a crucial role in avoiding misconceptions and preventing IBD women from remaining childless unnecessarily. Therefore, individuals with IBD who are contemplating parenthood should have access to a multidisciplinary team that can assist in dispelling myths and empower them to make informed choices during pregnancy and lactation. This support should be guided by the latest evidence and offer current recommendations, taking into account the emergence of a wide range of new IBD therapeutics, with additional promising medications in development (63).

### 2.4 Fatigue

Fatigue is one of the most frequently reported symptoms by patients with IBD, highly associated with poor HRQoL and disability. Nearly 80% of patients with active disease (64) and 63.8% of those with inactive disease report fatigue and lack of energy as the most burdensome symptoms (65), even surpassing BU and diarrhea (66), which may contribute to a decrease in HRQoL. Apart from its impact on QoL, fatigue also has economic consequences on patients’ lives. A survey among the general population in the United States estimated an annual direct and indirect cost of 100 billion American dollars attributable to fatigue (67). Reduced ability to travel or engage in social life, presenteeism and absenteeism can result in significant indirect costs that are frequently not considered in the cost-impact of the disease, probably because healthcare professionals tend to focus their attention on IBD symptoms and overlook fatigue during outpatient consultations. Noteworthy, fatigue consists of three components: the perception of generalized weakness and quick fatigability, the reduced capacity to maintain activities and the mental fatigue resulting in a lack of concentration, emotional stability and memory (68).

**Figure 2:**
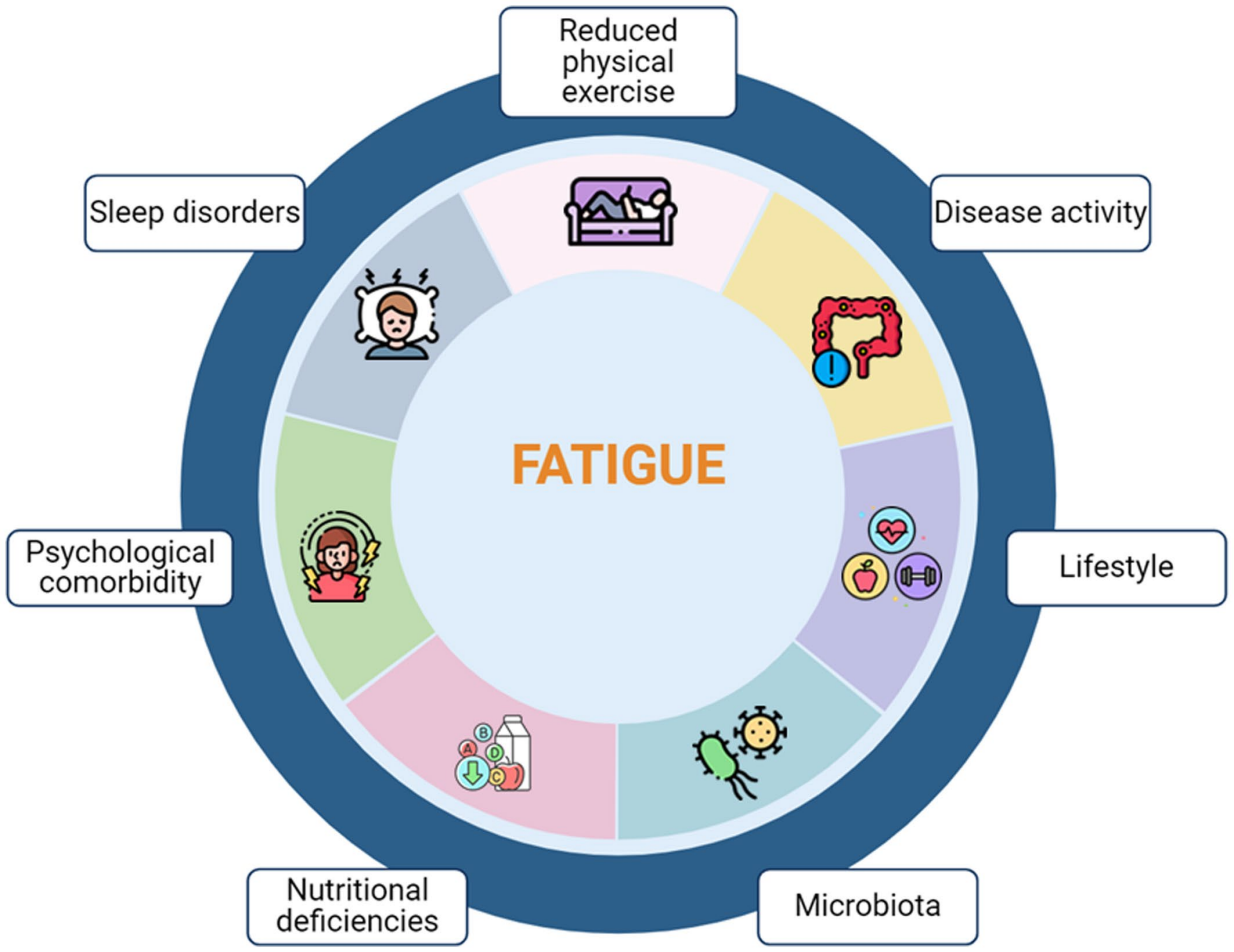
Multifactorial pathophysiology of Fatigue in IBD.

The pathogenesis of fatigue related to IBD, has yet to be determined. Several factors have been proposed to contribute to fatigue in IBD, including ongoing inflammation, microbiota composition, anemia, and micronutrient deficiencies and medical treatment (Figure 2). Firstly, inflammation plays a crucial role, as fatigue is more prevalent in patients with active disease than in those in remission (69). In cancer and chronic diseases such as IBD, the activation of the immune system triggers a pro-inflammatory state characterized by elevated concentrations of circulating cytokines. These cytokines reach the brain, inducing symptoms resembling sickness behavior, and the muscles, impairing their function and exacerbating fatigue. Indeed, certain single-nucleotide polymorphisms (SNPs) in pro-inflammatory cytokines have been found to be independently associated with higher fatigue scores, thus supporting the hypothesis of “cytokine-mediated sickness (70). Furthermore, fatigue and anxiety resulting from a pro-inflammatory state may evolve into mood disorders such as depression (71), which further exacerbates fatigue in a positive feedback mechanism. However, several studies have indicated that psychological factors are associated with fatigue in IBD patients independently of disease activity and inflammatory marker levels (72). Conditions such as depression or anxiety, which are prevalent in IBD patients at rates of 27 and 32%, respectively, (73), are strongly associated with fatigue. The overlap in symptomatology could result in their coexistence, including feelings of low energy and motivation, disrupted sleep patterns, and difficulty concentrating, alongside shared biological mechanisms. Besides, systemic inflammation may influence brain functioning through modifications in cerebral perfusion (74, 75), with some studies suggesting changes in intramembrane signaling (76) that could contribute to fatigue. Notably, patients experiencing fatigue were found to have reduced concentrations of glutamate and glutamine (77), both of which play pivotal roles in various brain functions, including mood regulation and energy metabolism.

Sleep disturbances have often been linked to most mental health conditions, including anxiety, depression and fatigue. Up to 82% of patients with IBD encounter substandard sleep quality, characterized by disturbances like disrupted sleep and nocturnal awakenings (78, 79). This pattern persists even during disease remission, where the prevalence of sleep disorders ranges from 47 to 51% (80). In a recent cross-sectional survey from the GETAID (81) fatigue was influenced by sleep disturbances. They observed a high prevalence of sleep disturbances (sleep subscore >5) and severe sleep disturbances (emotion subscore >7) in 47.0 and 30.9% of patients, respectively. Patients with active IBD had more sleep disturbances than those without active IBD (56.0% vs. 38.6%, *p* < 0.001). Nonetheless, poor sleep quality might increase the risk of relapse (77), worsening inflammation and thus fatigue itself.

Furthermore, nutritional deficiencies especially iron deficiency and anemia can cause fatigue in patients with IBD, regardless of disease activity (82). Besides, other nutrient deficiencies can occur in IBD patients due to decreased intake or reduced absorption, such as vitamin B12 and D deficiencies (83–85).

In patients with active IBD, effective medical management has been observed to potentially ameliorate or alleviate fatigue. Conversely, it has been noted that certain medications themselves can also induce fatigue (83–86). It remains unclear whether heightened fatigue is directly attributable to the drugs or is instead linked to a more severe disease necessitating the use of these medications. If fatigue persists despite disease control, a screen for other conditions, nutritional deficiencies (such as iron and vitamins) and concomitant infectious diseases (such as CMV, EBV and toxoplasmosis) should be performed. Ultimately, psychotherapeutic approaches such as cognitive-behavioral therapy and solution-focused therapy should be taken into consideration. In certain situations, physical activity and exercise may prove beneficial in alleviating fatigue and improving immune function, mood, and muscle strength. A potential mechanism involved in the regulation of mood, behavior, and cognitive function is the triptofan-to-serotonin pathway as dysregulation has been reported in conditions with heightened fatigue perception. A recent multicenter randomized controlled trial has evaluated the effect of 5-hydroxytryptophan supplementation on fatigue in patients with inactive IBD. However, despite a significant increase in serum 5-hydroxytryptophan and serotonin levels, oral 5- hydroxytryptophan did not modulate IBD-related fatigue better than placebo (87).

Finally, a growing body of evidence shows that reduced gut-microbial diversity (“gut dysbiosis”) by increasing the permeability of the gut barrier (“leaky gut”) has been associated with several mental disorders and represents the key clinical feature of chronic fatigue (82, 88).

However, given that fatigue is self-reported and heterogeneous, various questionnaires have been developed for diagnosis. While unidimensional scales offer easy quantification of fatigue, a multidimensional approach could provide a more comprehensive assessment by identifying the severity of fatigue and its impact on QoL. The strong correlation between fatigue and a broad spectrum of patient-reported outcome measures underscores the significance of fatigue-specific treatments in enhancing patients’ overall well-being. Nonetheless, interventions targeting fatigue in IBD remain limited; a recent prospective case series has shown that modafinil, blocking dopamine reuptake transporters, significantly improved IBD fatigue, suggesting that deficiency of centrally available dopamine may be a crucial cause of IBD fatigue (89). However, raising awareness and evaluating fatigue risk factors represent crucial initial steps in comprehending the pathophysiological mechanisms. These efforts may pave the way for the development of novel management approaches.

## 3 Conclusion

For a long time patients and physicians were not aligned on treatment goals and there were significant discrepancies regarding the definition of remission between them, which may affect expectations and clinical outcomes. However, in recent years, the target is not only the control of inflammation but also physical and emotional well-being. Sexual dysfunction, infertility, fatigue and BU are important areas that may require greater attention and are often overlooked, yet they all significantly impact disability and QoL of IBD patients. In this context, a holistic approach, including patient education and emotional and psychosocial support, should be implemented to enhance patient-physician communication. In this review, we will highlight the burden of disability-related symptoms and issues and the importance of acknowledging them and suggest ways of assessing them in clinical practice. However, further research is warranted to evaluate psychological and behavioral interventions in patients with IBD and to develop responsive and reliable patient-reported outcome measures. These measures are essential for demonstrating treatment benefits in clinical trials and enhancing communication in clinical practice, with the goal of improving overall QoL and reducing disability. A holistic approach with a patient-centric clinical care model is expected to change the way we practice medicine.

## Author contributions

OMN: Conceptualization, Writing – original draft, Writing – review & editing. GC: Investigation, Writing – original draft, Writing – review & editing. ALM: Investigation, Writing – original draft, Writing – review & editing. RC: Writing – review & editing. AT: Writing – review & editing. FC: Supervision, Writing – review & editing.
